# *Faecalibacterium* diversity in dairy cow milk

**DOI:** 10.1371/journal.pone.0221055

**Published:** 2019-08-16

**Authors:** Keith W. Savin, Jody Zawadzki, Martin J. Auldist, Jianghui Wang, Doris Ram, Simone Rochfort, Benjamin G. Cocks

**Affiliations:** 1 AgriBio Centre, Agriculture Victoria Research, Bundoora, Victoria, Australia; 2 Agriculture Victoria Research, Ellinbank, Victoria, Australia; University of Minnesota Twin Cities, UNITED STATES

## Abstract

The bacterial species, *Faecalibacterium prausnitzii*, beneficial to humans and animals and found in mammalian and avian gut, is also occasionally found in dairy cow milk. It is one of the butyrate-producing bacteria of the colon, has anti-inflammatory properties and its abundance in the gut is negatively correlated with obesity in humans. Several strains differing in their functional capability, have been identified. It is important therefore, milk being a potential source of *F*. *prausnitzii* as a novel probiotic, to investigate the diversity of this species in bovine milk. Using 16s rRNA gene amplicons we find 292 different dereplicated *Faecalibacterium*-related amplicons in a herd of 21 dairy cows. The distribution of the 20 most abundant amplicons with >97% identity to a Greengenes OTU varies from cow to cow. Clustering of the 292 pooled sequences from all cows at 99.6% identity finds 4 likely *Faecalibacterium* phylotypes with >98.5% identity to an *F*. *prausnitzii* reference sequence. Sequence alignment and phylogenetic analysis shows these phylotypes are distinct from 34 other species from the *Ruminococcaceae* family and displaying the sequence clusters as a network illustrates how each cluster is composed of sequences from multiple cows. We conclude there are several phylotypes of *Faecalibacterium prausnitzii* (the only species so far defined for the genus) in this dairy herd with cows being inoculated with a mixture of several strains from a common source. We conclude that not only can Faecalibacterium be detected in dairy cow milk (as noted by others) but that there exist multiple different strains in the milk of a dairy herd. Therefore milk, as an alternative to faeces, offers the opportunity of discovering new strains with potential probiotic application.

## Introduction

While dairy cow milk is an important food source for many humans and is known to contain many species of bacteria[1–3], until recently its microbial population had not received the degree of detailed attention paid to microbiomes such as in the human gut. We know from earlier studies that milk from humans and farm ruminants is dominated by *Proteobacteria*, *Firmicutes*, *Actinobacteria* and *Bacteroidetes* phyla [4–6]. The more recent development of high throughput sequencing of microbial 16s rRNA gene variable regions now enables a detailed description of milk microbiomes such as that found in human [7, 8] and dairy milk[1, 5, 9].

Several bacterial genera are thought to be beneficial for human health. Here we focus on *Faecalibacterium* in milk. First discovered in human faeces [10, 11] *Faecalibacterium prausnitzii* is one of the butyrate producers in the colon and has important immune and metabolic functions in relation to inflammation [12, 13] and possibly obesity [14–16]. It is seen in agricultural species such as cows, pigs and poultry, where it may be a source of new *F*. *prausnitzii* strains [17,18,21] as well as being important for their health. Higher *Faecalibacterium* abundance in faeces has been linked to higher weight gain and less frequent diarrhea in bovine calves[22]. The strains of Faecalibacterium present in the faeces of calves and pigs have been studied with the aim of developing a probiotic for farm animals[21]. Subsequently it was found that oral administration of these bacteria to preweaned dairy heifers did indeed improve weight gain and reduce diarrhea occurrence[23]. Associations have been noted between Faecalibacterium abundance in caecum and faeces versus health and growth of chickens[24–26].This species has been seen in some analyses of cow’s milk[27, 28] and human milk[29, 30], but not in others[31–33]. *F*. *prausnitzii* is the only species so far defined in the *Faecalibacterium* genus, making it more amenable to identification using 16s rRNA amplicon sequencing as this gene can distinguish it from its relatives in other genera of the Ruminococcaceae family[34].

Different strains of *F*. *prausnitzii* have been identified in the human gut and may differ in their functional role, especially in relation to anti-inflammatory properties[34–38]. We considered it important therefore, milk being a potential source of *F*. *prausnitzii* in the human diet, to investigate the diversity of the phylotypes of this genus in dairy cow milk.

Milk was originally thought to be sterile until contaminated by microbes from the local soil, faeces, skin or water supply. Many of the hundreds of microbial species found in raw dairy cow milk within the mammary gland [5] may indeed have come from the local dairy farm microbial ecosystem, yet may serve an important role in populating the gut of newborn animals with representatives of their local microbial environment, in addition to the microbial species acquired from the mother’s microbiome as has been seen in humans[39] where the possible role of milk has been discussed with regard to delivering microbes to the newborn human gut [40]. The importance of gut bacteria in human health is now undeniable [41] and presumably is just as important in the bovine gut, even before considering bovine milk as a major human food source. Some bacterial species discovered in human and cow’s milk and thought to be beneficial to human health, such as members of the *Lactobacillus* genus, have been used as probiotics for deliberate addition to the human diet [5, 42]. Other species such as *Faecalibacterium prausnitzii* may also offer human health benefits if included in the human diet as a probiotic. Using 16s ribosomal RNA gene variable region 4 (V4) DNA sequences we analysed the microbiome of milk samples from 21 cows from a single herd, examining the diversity of *Faecalibacterium* 16s V4 sequences from cow to cow and within the herd as a whole.

## Materials and methods

### Ethics approval and consent to participate

All cows were maintained in the research herd at the Agriculture Victoria Ellinbank Centre, 1301 Hazeldean Road, Ellinbank, Victoria, Australia. All experiments were conducted in accordance with the Australian Code of Practice for the Care and Use of Animals for Scientific Purposes (National Health and Medical Research Council 2004). Approval to proceed was obtained from the Department of Environment and Primary Industries Agricultural Research and Extension Animal Ethics Committee. No anesthesia, euthanasia, or any kind of animal sacrifice was part of the study. The milk was collected from the regular daily milking cycle of the herd. As there was no animal intervention/experimentation involved, other than routine milk collection, only general approval from the Department of Environment and Primary Industries Agricultural Research and Extension Animal Ethics Committee was required.

### Cows, milk microbes and DNA sequencing

Cows were husbanded and milk samples collected as described[43]. All cows were maintained in the research herd at the Agriculture Victoria Ellinbank Centre, and the experimentation was conducted in accordance with the Australian Code of Practice for the Care and Use of Animals for Scientific Purposes. Briefly, cows were fed on pasture and grains (S1 Table). The cows in this study were the first of the herd to enter the milking parlour. The milking procedure was designed to prevent carry-over of milk from one cow's sample to another. Each cow was milked from a separate disinfected station twice daily at 0600h and 1500h (MM25; DeLaval International, Tumba, Sweden). Milk from each cow was collected directly into a disinfected stainless steel milk can from which a sub-sample of 100ml was then taken onto ice. The 0600h milk sample was pooled with the previous day's 1500h collection sample which had been stored on ice. From the 200ml of pooled morning plus afternoon samples a 50ml aliquot was transported and stored at 4°C before DNA extraction within 24 hours.

An enriched fraction of microbial DNA was prepared based on a published method[44]. Briefly, Triton-X100 detergent was added to the milk to 1% v/v to lyse bovine cells and dissolve fat globules followed by centrifugation at 3220g for 30 minutes and the pellet suspended in phosphate-buffered saline. Casein micelles were solubilised and removed from the pelleted material by the addition of EDTA to 30mM, further centrifugation and saline washes. During development of the procedure, DNA from the pellet was analysed by PCR, using 16s v4 primers, to test for the presence of bacterial DNA sequences. This identified the pellet as being enriched for bacteria relative to the supernatant. Microbe-enriched genomic DNA was extracted from the pellet using a QIAamp DNA Mini Kit (Qiagen).

The no-DNA controls were samples with the same detergent, buffer, DNA polymerase enzyme, etc, but with no DNA from milk. They were processed in parallel with the DNA from milk. The milk DNA samples and 2 no-DNA controls were all subjected to the same PCR reactions and DNA sequencing together as a batch. To make the reaction volumes the same, the milk DNA solution was replaced with water in the controls.

Variable region 4 from the prokaryote 16S rRNA gene was amplified by polymerase chain reaction (PCR) using the F515/R806 primers (5’-GTGCCAGCMGCCGCGGTAA-3’/ 5’ -GGACTACHVGGGTWTCTAAT-3’) and associated published method[45], using Phusion DNA polymerase (ThermoFisher Scientific). The Illumina MiSeq system was used to generate DNA sequence data from the PCR fragments (251x 2 cycles, paired-end sequencing), according to the manufacturer’s instructions. The fastq files generated are available at Figshare [46] including those from no-DNA negative control PCR reactions.

### Processing of 16s V4 amplicon DNA sequences

DNA sequence fastq files from MiSeq paired end (PE) sequencing were quality filtered using Trimmomatic software[47]. Quality score by base position plots of raw and filtered sequence data were generated by fastQC [48] and examined manually. Processing of fastq files, containing overlapping 5’ or 3’ end sequences, was carried out using basic unix utilities and the PANDAseq program [49] for assembly of paired-end sequences. Assembled sequence pairs were retained if they contained the 5’ and 3’ PCR primer sequences at their ends (using regular expressions based on the above sequences). Primer sequences were then removed from the sequence ends and sequences rejected if an ambiguous base (an N character) was present anywhere in the sequence. Unix utilities such as grep, gawk, sort and uniq were then used to group and count replicate (100% identical) sequences, creating files of de-replicated sequences in fasta format.

MegaBLAST [50] was used to compare the de-replicated sequences to the Greengenes ribosomal RNA gene sequence database (August 2013 version, ftp://greengenes.microbio.me/greengenes_release/current), the Genbank collection of microbial 16s ribosomal RNA gene sequences and to the prokaryote genome database from ENSEMBL. Unix utilities were used to parse the BLAST output file, extracting the query name, count and percent identity to a Greengenes or other database sequence as required. BLAST parameters and result parsing were set such that sequence alignments must be full length (253 bases) and of at least 97% sequence identity, otherwise being classified as no hits or unassigned. This process also eliminates chimeric sequences except potentially where a sequence in the database is itself a chimera. Python was used to construct sample by taxon abundance counts tables from the BLAST results. Taxonomies were initially assigned based on the Greengenes rRNA OTU database. Subsequently, for clustered sequences, taxa were also assigned using the 16s rRNA and prokaryote genome databases (see below).

### Analysis of 16s V4 amplicons

A rarefaction plot (S1 Fig) was produced using increasing multiples of 10,000 16s amplicons derived from a bulk milk sample (pooled milk from 5 cows). Using the unix utility shuf, batches of sequences were randomly selected from PANDAseq-assembled pairs of MiSeq PE sequences (not de-replicated). Each batch of sequences was clustered to 97% identity using uparse [51], 99.6% identity using swarm[52] or aligned to sequences in the Greengenes database using MegaBLAST. The number of clusters or bacterial or archaeal species identified to at least 97% identity was counted. This included those taxa not annotated down to the species level. Greengenes species-level OTU found in the PCR negative controls were ignored in subsequent analyses.

Shannon diversity index and Chao1 species richness were calculated using algorithms in the R libraries vegan and fossil respectively. See S2 Table for a summary of milk sample sequencing results.

R was used for plotting abundance data as barcharts and for network visualisation of sequence clusters generated by swarm. Base R functions were used, together with functions from the vegan, permute, fossil, lattice and igraph libraries.

The clustering of de-replicated Pandaseq-assembled sequences from the whole herd was carried out using swarm version 2.1.9 with the parameters–f–t 4. Swarm clustering was applied only to those amplicon sequences identified as *Faecalibacterium* by comparison to one or other of the 3 databases (Greengenes, Genbank 16s or ENSEMBL prokaryote genomes at >97% sequence identity). Clusters (swarms) were visualised with a network plot using the R igraph library. The igraph input files (edges list, node attributes and legend, see figshare[44]) were manually generated using the swarm2 output files that contain the swarm sequence membership and structure. In the network figure the edges between centroids and surrounding amplicons represent sequence differences of 0 or 1 base (ie >99% identity).

A phylogenetic comparison of *Faecalibacterium*-related sequence clusters with other members of the *Ruminococcaceae* family used muscle[53] to align the 6 most abundant swarm centroid DNA sequences and 16s V4 sequences from 35 different *Ruminococcaceae* species, including 3 different *Faecalibacterium prausnitzii* strains. The resulting Neighbour-Joining tree[54–56] was visualised using MegaX[57].

## Results

### Amplicon sequences

Milk samples were collected from each of 21 Holstein dairy cows in mid lactation. The populations of bacteria and archaea harvested from each milk sample yielded a mean of 262822 pairs of MiSeq DNA sequences, ranging from 128528 to 503917, (S2 Table). Each milk sample yielded an average 61396 different unique (de-replicated) Pandaseq assembled 16s variable region 4 sequences of 253 bases in length. Each of these unique sequences represents a set of replicate sequences with abundances varying from one to many thousands, depending on which taxon they derive from, the taxon’s abundance and the copy number of the 16s rRNA gene in the relevant species.

A rarefaction analysis of sequence data from a bulk milk sample (containing pooled milk from 5 cows) was carried out to test if sufficient sequence reads were being generated for detection of taxa present. The counts of assembled read pairs, clusters and taxa were plotted and can be seen in S1 Fig. The 16s sequences were clustered using swarm and Uparse to 99.6% and 97% sequence identity respectively. The Uparse-clustered sequence counts were also plotted after removing singletons. A significant drop in sequence clusters was seen with the latter. While the number of assembled read pairs and clusters continue to increase, the number of different taxa approaches a ceiling, especially when species present at less than 0.1% abundance are removed. Beyond 100,000 unique 16s sequences, the number of new taxa discovered was negligible. All our MiSeq sequence libraries generated at least 120,000 read pairs, sufficient sequence reads to allow us to identify all taxa present at above 0.1% abundance plus many rare taxa.

The Shannon species diversity index and Chao1 species richness measures were calculated using the species abundance counts from the MegaBLAST analysis of unique sequences. These diversity indices are both within the expected range for this type of microbiome, such as that seen in published human gut and milk analyses[27, 58–60]. These data can be seen in S2 Table.

### Microbiome content

**Fig 1:**
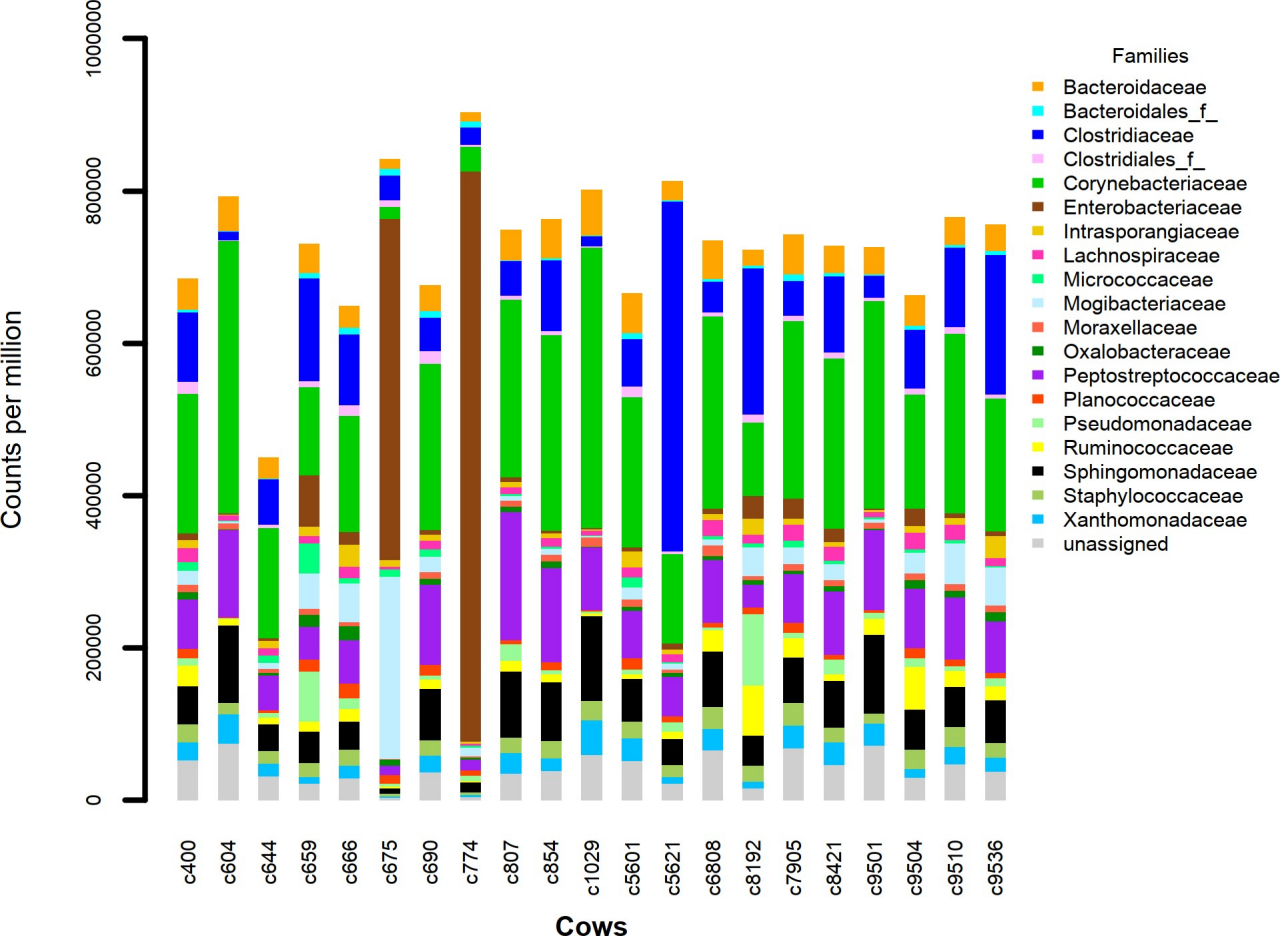
Stacked barplot of the 20 most abundant microbial families identified in 21 Holstein dairy cow milk samples, expressed as counts per million (cpm) 16s rRNA geneV4 amplicon sequences corresponding to the taxa in the Greengenes rRNA sequence database.

The dominant phyla in all 21 milk samples were the *Firmicutes*, *Proteobacteria*, *Bacteroidetes* and *Actinobacteria*. The bacterial families *Staphylococcaceae*, *Ruminococcaceae*, *Lachnospiraceae* and an undefined family of the *Clostridiales* order made up over 50% of the population (Fig 1). In 2 cows, *Pseudomonadaceae* comprised the majority of the bacteria. Fig 1 illustrates the limited variability of the 20 most abundant families (including unassigned) across the herd of 21 cows except for cows 675 and 774 which were kept indoors rather than in the open field. A visual assessment of Fig 1 indicates their milk microbiomes differ somewhat from the other cows at the family level. None of the 21 cows had been recently diagnosed with mastitis or treated with antibiotics in the month before sample collection. Based on the sequence annotation information in the Greengenes 16s rRNA database we found 35 different phyla encompassing 387 different microbial families in our collection of milk samples. At an abundance of at least 0.1% (1000cpm) 80 families were present. In general, 16s rRNA amplicons are not discriminatory at the species level of taxonomy. For some genera, many species are identical in the 16s V4 region meaning an inability to distinguish species from each other and leading to an underestimate of the species diversity and richness[19,20]. Our focus here is primarily on the presence of *Faecalibacterium* in dairy cow milk and the diversity of 16s rRNA gene sequences of this species. In the *Ruminococcaceae* family there is only a single species defined so far in the *Faecalibacterium* genus and this can be differentiated from the other *Ruminococcaceae* family members using the 16s rRNA gene V4 amplicon sequence (see below). This has been seen by others when comparing *Faecalibacterium* isolate 16s rRNA amplicon sequences to related species[21].

### Faecalibacterium phylotype distribution

**Fig 2:**
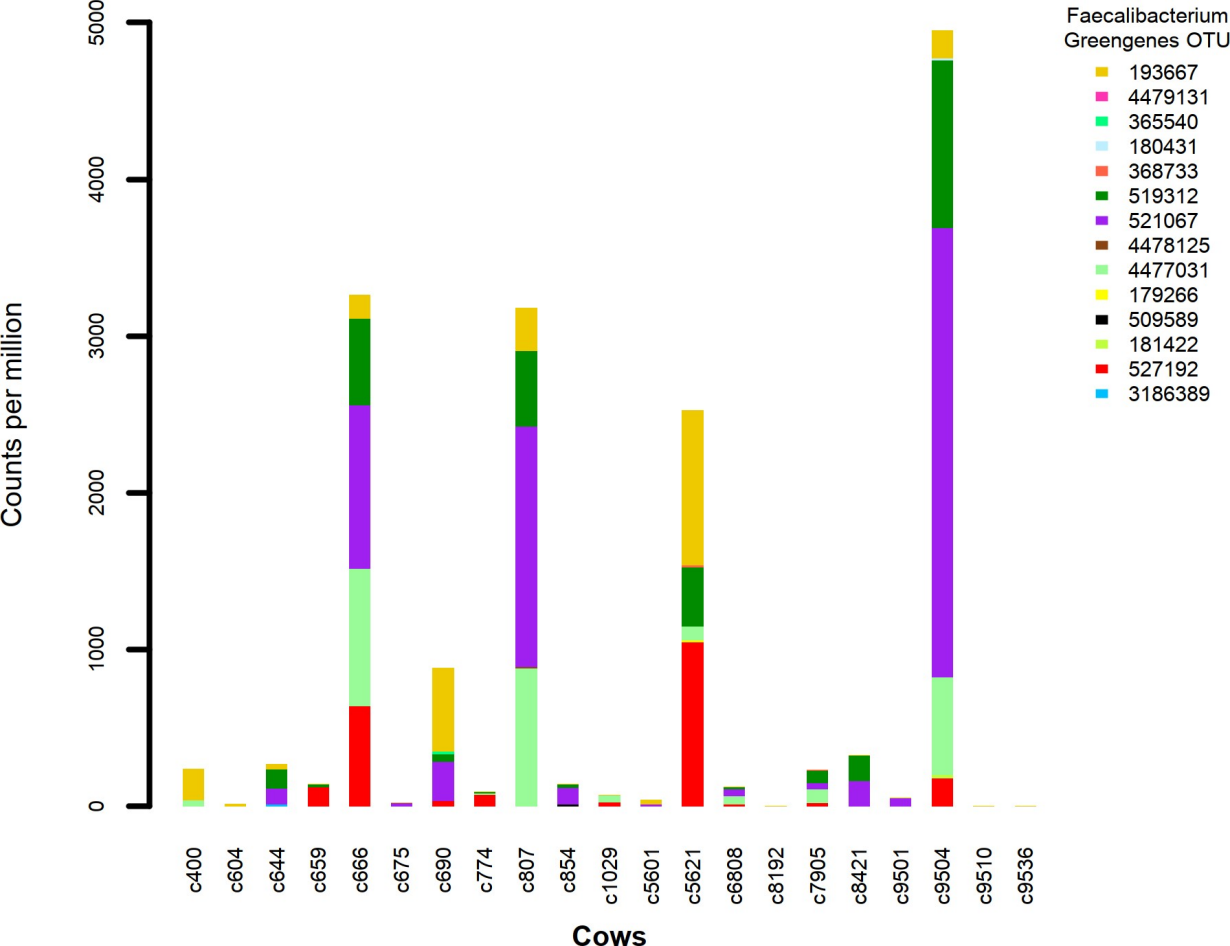
Stacked barplot of the abundance in cpm of 16s rRNA gene V4 amplicon sequences in each milk sample or cow, corresponding to the 14 identified *Faecalibacterium*-related Greengenes OTU.

Of the 1114 known Greengenes OTU representing *Faecalibacterium*, we identified 14 different OTU distributed across 18 of our 21 milk samples. Of the 292 unique (de-replicated) 16s V4 sequences identified as from Faecalibcterium by aligning to the Greengenes database, 282 are also identified as such by aligning to either the NCBI 16s rRNA gene or the NCBI representative prokaryote genome databases. A stacked bar chart of the identified Greengenes OTU (Fig 2) illustrates the highly uneven abundance of *F*. *prausnitzii* OTU across the collection of milk samples. None of the OTU are represented in every milk sample (ie every cow) and the more highly abundant OTU are confined to less than half the samples. A visual assessment of the plot indicates cows 666, 6808, 7905 and 9504 are somewhat similar in OTU abundance pattern, but little similarity is seen across the remainder of the cows.

**Fig 3:**
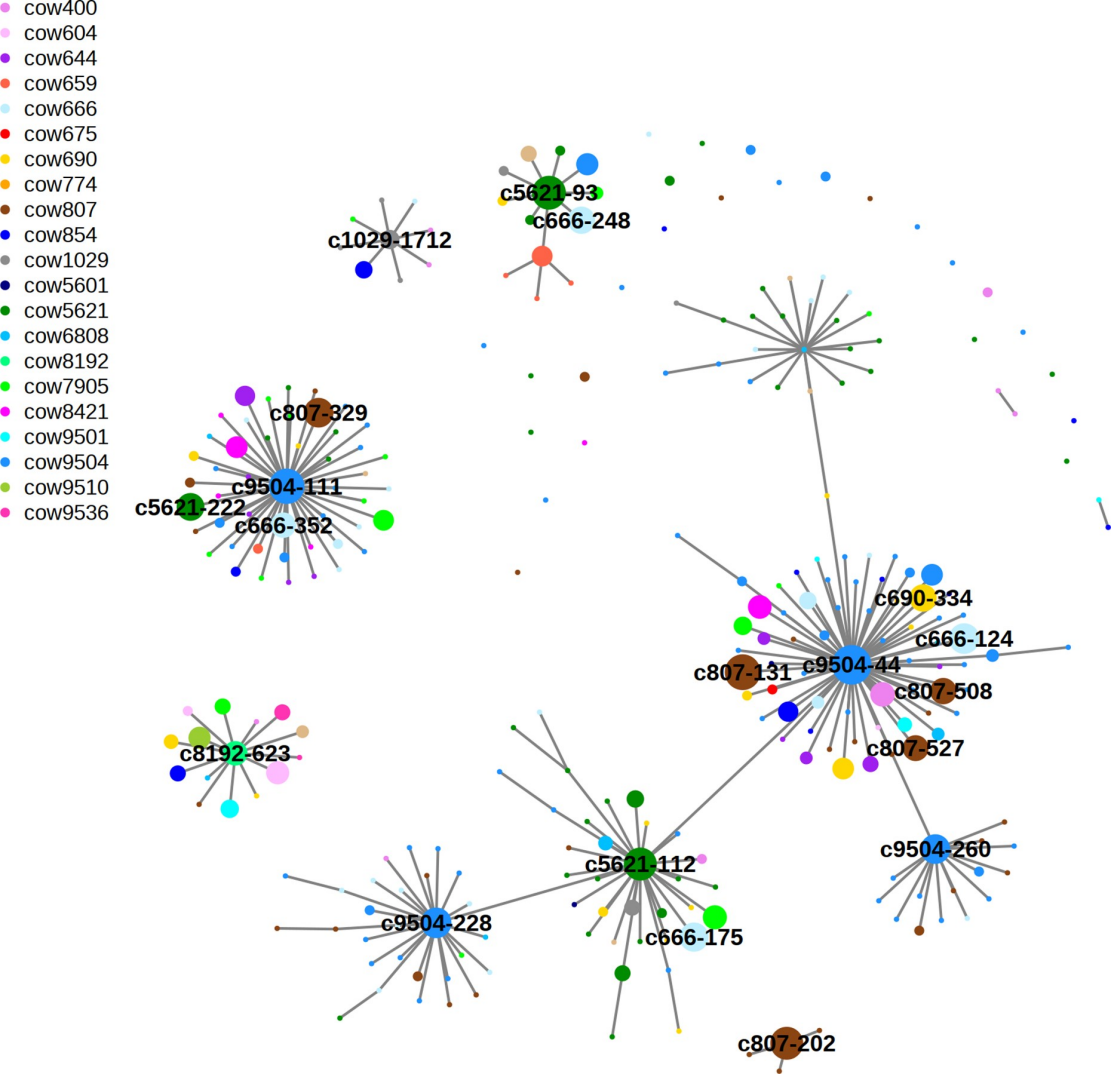
Network analysis of clustered *Faecalibacterium* 16s V4 sequences (centroids). Clustering used swarm2 where the difference between adjacent sequences (edges that connect centroids) within a cluster is a single base change (99.6% identity). Centroids are coloured according to cow and circle diameter is based on sequence abundance (d = 1+log(cpm)). Edge length is for visual presentation only and does not reflect degree of sequence identity.

To examine the diversity of *Faecalibacterium* 16s RNA gene sequence variants, or phylotypes, across the herd we pooled and clustered the 292 dereplicated *Faecalibacterium*-related amplicon sequences (based on Greengenes database MegaBLAST results) at a high level of DNA sequence identity using swarm. This software clusters sequences around a central seed sequence or centroid forming hierarchical levels based on differences of only a single nucleotide. The network diagram in Fig 3 illustrates the connections between cows and sequences.

Each central sequence (centroid) of a cluster is surrounded by its close relatives and is represented by a circular node. The node colour represents the host cow the sequence was found in and the node diameter its abundance in that cow. All edges represent differences of 0 or 1 nucleotide (99.6% identity). The authors of swarm refer to the clusters as “swarms”. Most swarms contain different coloured circles indicating amplicons from several different cows make up the swarm. These single base differences could be polymorphisms (SNPs) generated in vivo during bacterial growth or laboratory-generated PCR or sequencing errors. Several of the larger swarms have few surrounding single base mutants. This suggests most of the single base differences are likely to be biological rather than artefactual as all the DNA samples were processed together and error rates from PCR or sequencing should be similar across an experiment. A summary of the MegaBLAST analysis results, plus the DNA sequence, for the most abundant 6 centroids compared to the NCBI Microbial 16s database can be seen in S3 Table.

### Milk *Faecalibacterium* versus reference strains and other *Ruminococcaceae*

**Fig 4:**
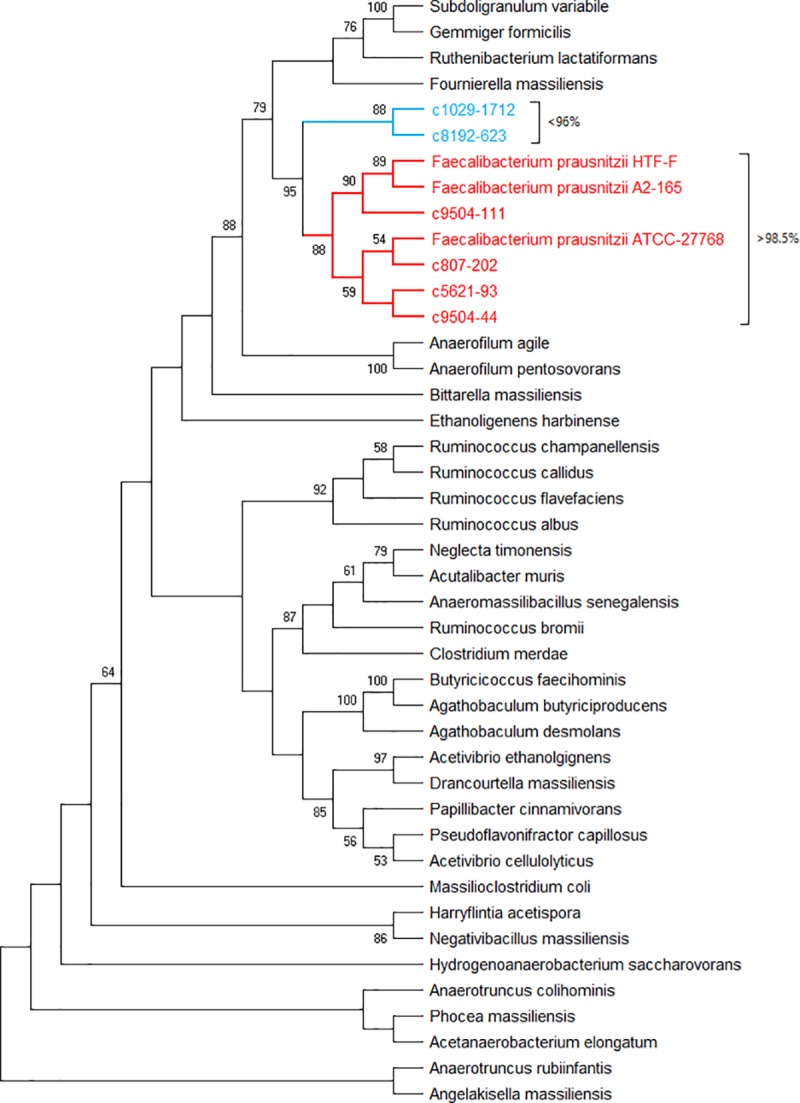
Evolutionary relationships of taxa: Cladogram of Ruminococcaceae 16s v4 sequences. The evolutionary history was inferred using the Neighbor-Joining method. The optimal tree with the sum of branch length = 1.5 is shown. The percentage of replicate trees in which the associated taxa clustered together in the bootstrap test (500 replicates) are shown next to the branches if >50%. The tree is drawn to scale, with branch lengths in the same units as those of the evolutionary distances used to infer the phylogenetic tree (number of base substitutions per site). The distances were computed using the Maximum Composite Likelihood method. This analysis involved 43 nucleotide sequences. Evolutionary analyses were conducted in MEGA X. Centroids with >98.5% sequence identity to the reference strain F. prausnitzii ATCC-27768 are shown in red, those with <96% identity to the reference are in blue.

Finally, to test if the *Faecalibacterium*-related centroid sequences found in our milk samples can be differentiated from other *Ruminococcaceae* species using the 16s rRNA gene V4 amplicon, we used muscle to align the 6 most abundant centroids to 3 reference strains of *F*. *prausnitzii* plus 16s rRNA gene V4 sequences from 34 different *Ruminococcaceae* species. Fig 4 illustrates a neighbour-joining phylogenetic tree (cladogram) produced from the muscle alignment. The 4 centroids with >98.5% sequence identity to the sequence from *F*. *prausnitzii* strain ATCC 27768 are seen to occupy a clade of closely related sequences. The 2 centroids with <96% identity to the same reference strain form a neighbouring clade. Considering that 97% is usually taken to be the minimum for species identity (in cases where species can be differentiated using this amplicon), these 2 centroids may derive from an as yet unidentified species. In addition, if the milk centroid sequences are clustered with the v4 sequences of 280 Faecalibacterium and 7 other Ruminococcaceae family members, the centroids can be seen in 5 clades containing human or bovine Faecalibacterium sequences (see S2 Fig). When these 295 16s v4 sequences, including 7 from non-Faecalibacterial species, are clustered to the 100% identity level, they are reduced to 17 different Faecalibacterium and 7 other Ruminococcaceae sequences forming 7 Faecalibacterium plus the other Ruminococcaceae (see S3 Fig).

## Discussion

For bovine milk, the major bacterial phyla we observe, as have others, are consistently seen across the cow herd. The most abundant phyla in our milk samples are the *Firmicutes*, *Proteobacteria*, *Bacteroidetes* and *Actinobacteria* phyla. The most likely contaminant of milk (if we are to consider them as contaminants) would be the microbes of faeces. In the colon this is dominated by *Firmicutes* and *Bacteroidetes*, but *Proteobacteria* and *Actinobacteria* make up significant proportions of the faecal microbiome after only a few days exposure to the field environment [61]. On the other hand, at least some milk bacteria may have their origins in the gut via the proposed entero-mammary pathway[62, 63] or the bloodstream[64]. In our milk samples 35 different phyla can be detected containing 387 different families. Only 80 families are found with an abundance of at least 0.1%(1000cpm) in at least 5 cows and these are from only 12 phyla. The figure showing the most abundant families indicates the pattern is similar across 19 of the 21 cows. The rarefaction, diversity and species richness analyses, based on the species annotation of the Greengenes database, indicate we are sequencing to a sufficient depth and identifying species at a diversity comparable to work on other microbiomes such as gut or human milk[27, 58–60] (remembering that the 16s amplicon method will give an underestimate of the species diversity).

Here we focus only on one genus, *Faecalibacterium*, originally discovered in human faeces and known to inhabit the ileum, colon and cecum of mammals[10, 65] and the cecum of birds[66]. In humans and model species such as mice, *F*. *prausnitzii* studies have focussed on its importance in generating butyrate and on regulating the host immune system. While butyrate generated by *F*. *prausnitzii* and other bacterial species is an important energy source for colonic epithelial cells, it also has multiple epigenetic effects likely due to its activity as an inhibitor of histone deacetylase[67], with outcomes as diverse as regulating metabolism[68, 69], modulating the immune system, especially T lymphocyte development in relation to inflammatory bowel diseases [12, 70–72] or to suppressing the growth of tumour cells[73, 74]. The influence of *F*. *prausnitzii* on the gut-associated immune system is likely also mediated by more complex molecules secreted by the bacterial cells such as the Microbial Anti-inflammatory Molecule (MAM) from strain A2-165 or the polymeric matrix protein from strain HTF-F. These may be responsible for the differing anti-inflammatory behaviour of the latter 2 strains[35, 37].

Some recent research has set out to identify new strains of *F*. *prausnitzii* from human gut as a potential source of novel probiotics for improving general human health and more specifically for treating inflammatory bowel diseases[36, 38]. Functional comparisons of known strains[37, 75] suggests it will be important in the future to group strains based on function as well as phylogeny. More recently has come the suggestion *prausnitzii* may not be the only species in the *Faecalibacterium* genus[76].

By clustering with swarm we reduce 292 different de-replicated *Faecalibacterium*-related amplicon sequences from the whole herd of 21 cows to 35 centroid sequences. Each centroid is the most abundant member of a cluster, or swarm, also containing close relatives, each with a different single nucleotide polymorphism (including single base insertions and deletions). A centroid can be a single sequence or can represent many hundreds of 100% identical sequences.

Examination of the amplicon centroids in the form of a network illustrates how each swarm is actually composed of sequences from several cows. Were the network clusters each from single cows we might conclude that each cow had its own phylotypes, or perhaps strains, of the bacterium which had occupied their mammary gland over the long term, generating polymorphisms over time. However, this is not the case. The members of each network cluster come from different cows, suggesting inoculation of mammary glands from a single source in the shared environment of the dairy.

Finally, there is an argument that the 16s V4 amplicon is unable to differentiate bacteria at the species, or even the genus level. This can be seen with genera such as Pseudomonas, where the 16s v4 sequence is identical across many Pseudomonas species. To test this with *Faecalibacterium* we aligned the 16s rRNA gene v4 region from the 6 most abundant centroids, 3 reference strains of *Faecalibacterium prausnitzii* and 34 other species from the *Ruminococcaceae* family and plotted a phylogenetic tree from the result. The *Faecalibacteria* and the centroids share a clade which is distinct from the other Ruminococcaceae and is itself split into 2 subclades based on degree of sequence identity.

These 16s V4 centroids are best referred to as representing phylotypes, not strains. Strains are unlikely to be differentiated by small amplicon sequences, even in the *Faecalibacterium*. Nevertheless, the presence of multiple different phylotypes implies the potential existence of multiple strains of *Faecalibacterium prausnitzii*. A deeper analysis of *Faecalibacterium* strains could perhaps be achieved by amplifying and sequencing the genes encoding enzymes for butyrate synthesis [77] or the anti-inflammatory protein MAM[35]. Genome analyses such as that recently published by Benevides *et al* [36] will help in this regard, especially if combined with the isolation by culturing of new *Faecalibacterium* strains followed by functional analysis.

## Conclusion

A bacterial genus beneficial to humans, *Faecalibacterium*, although rare in milk, was seen to vary in abundance more than 100 fold between 21 cows, with each cow appearing to have a different pattern of 16s rRNA amplicon phylotype abundance.

The pattern of Greengenes OTU distribution and the network of *Faecalibacterium* sequence clusters (swarms) suggests each cow may have its own characteristic *Faecalibacterium* population. The data indicate multiple strains or perhaps multiple as yet undefined *Faecalibacterium* species, are likely present in the milk samples, perhaps deriving from a source in the dairy farm environment such as faeces, rumen, soil, water, animal or human skin or milking equipment.

Phylogenetic analysis of aligned sequences confirms 6 of the 8 most abundant phylotypes can be differentiated from those of other *Ruminococcaceae* genera and are closely related to 3 different known *Faecalibacterium prausnitzii* strains isolated from human and bovine faeces. Clustering of pooled *Faecalibacterium*-related 16s v4 DNA sequences from the whole herd indicates most are present as members of 5 clusters, with each cow contributing sequences to at least 3 clusters. A network diagram of the sequence clusters shows most are contributed to by multiple cows, suggesting a common source for the bacteria, with subsequent differential growth or a filtering process selecting for some strains over others in each cow. A simpler hypothesis would invoke random acquisition of a limited number of bacterial cells from a common unknown source.

DNA sequence data from the various dairy farm microbiomes (milk, faeces, skin, water, equipment) may help identify the source of various milk bacteria, especially if closed circle sequencing of long reads were employed for example, greatly increasing the accuracy of species or perhaps strain identification. Nevertheless these data indicate many cows in a dairy herd are likely to carry multiple different members of the *Faecalibacterium* genus in their milk. The next step is for strains from milk to be isolated in culture and undergo functional analysis. We expect that milk, as an alternative to faeces, offers the opportunity of discovering new *Faecalibacterium prausnitzii* strains with potential probiotic application.

## Supporting information

S1 Fig Rarefaction plot of increasing numbers of unique 16s sequences (Uniques, after Pandaseq assembly of paired reads). Increasingly larger groups of randomly selected sequences were clustered with swarm (swarms, 99.6% identity), Uparse (Uparse97, 97% identity) or megaBLAST vs the Greengenes rRNA taxonomy database (Species, 97% identity). Also shown are counts after removal of singletons from Uparse clusters(Uparse97no1) and removal of low abundance(<0.1%, megaBlast) sequences from species(species.01%).
(TIF)

S2 Fig Evolutionary relationships of rRNA 16s gene v4 sequences: Phylogram of 295 *Ruminococcaceae* 16s v4 sequences. The evolutionary history was inferred using the Neighbor-Joining method after clustering the sequences with clustal Omega and plotting the tree after 1000 bootstrap replications. The type strain *F*. *prausnitzii* ATCC-27768 is shown in brown. *F*. *prausnitzii* sequences analysed were from human faeces (black), bovine faeces (magenta), duck faeces (green), milk centroids (red). Included were 7 sequences from non-*Faecalibacterium* members of the *Ruminococcaceae* family (blue).
(TIF)

S3 Fig Evolutionary relationships of rRNA 16s gene v4 sequences: Cladogram of 24 *Ruminococcaceae* 16s v4 sequences that resulted from dereplicating (clustering at 100% identity) the sequences used for S2 Fig. The evolutionary history was inferred using the Neighbor-Joining method after clustering the sequences with clustal Omega and plotting the tree after 100 bootstrap replications. The tree was additionally annotated to display the strains or species from which the dereplicated sequences (blue) derived. The names of the milk centroids are shown in magenta and the non-*Faecalibacterium* members of the *Ruminococcaceae* family are shown in red.
(TIF)

S1 Table Location and diet of cows. Nineteen of the 21 cows were kept outdoors in a field growing pasture(ryegrass). In addition they were each fed grains, silage and hay. Cows 675 and 774 were kept indoors and fed on grains and hay. Diet figures are daily intake (pasture is estimated).
(DOCX)

S2 Table Summary of sequencing and taxonomy analysis for each milk sample or cow. Read-pairs = total pairs of sequence reads generated by MiSeq; De-rep = number of unique 16s V4 sequences assembled by Pandaseq, filtered and de-replicated; OTU = number of different Greengenes OTU detected by MegaBlast with at least 97% identity and with >10 counts per million (cpm) abundance; Phyla = number of different phyla present at >10000cpm(1%) abundance; H = Shannon diversity Index; Chao1 = Chao1 species richness estimate. Shannon diversity Index and Chao1 species richness use the counts at species level as available in the August 2013 version of the Greengenes ribosomal RNA database, as are also the phyla and genera abundances.
(DOCX)

S3 Table Summary of blast analysis results for 6 most abundant swarm centroids. *Faecalibacterium*-related (from analysis using the Greengenes database) 16s v4 sequences, clustered using swarm2(see Fig 3) were compared to the Genbank Microbial 16s rRNA gene sequences database using megaBlast. Listed are results from the 6 most abundant centroid sequences, including centroid DNA sequence, Genbank accessions, expect values and sequence identities.
(DOCX)

## Abbreviations

OUT: Operational Taxonomic Unit
