# Supplementary figures and images for: Faecalibacterium diversity in dairy cow milk
Source: PLoS One. 2019 Aug 16;14(8):e0221055. doi: 10.1371/journal.pone.0221055 (PMC6697359; doi:10.1371/journal.pone.0221055)

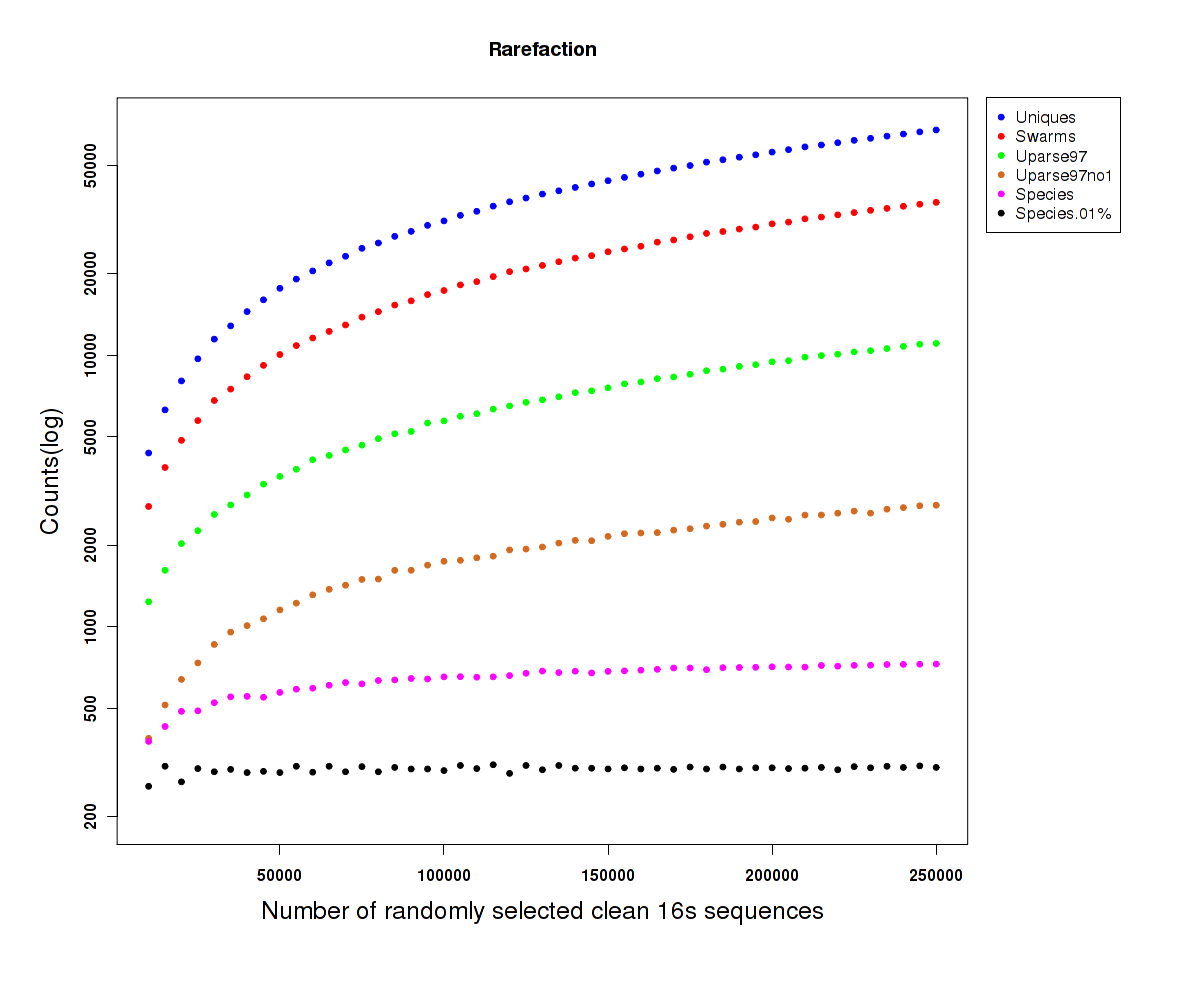

Supplement: S1 Fig — Increasingly larger groups of randomly selected sequences were clustered with swarm (swarms, 99.6% identity), Uparse (Uparse97, 97% identity) or megaBLAST vs the Greengenes rRNA taxonomy database (Species, 97% identity). Also shown are counts after removal of singletons from Uparse clusters(Uparse97no1) and removal of low abundance(<0.1%, megaBlast) sequences from species(species.01%). (TIF) [file pone.0221055.s001.tif]

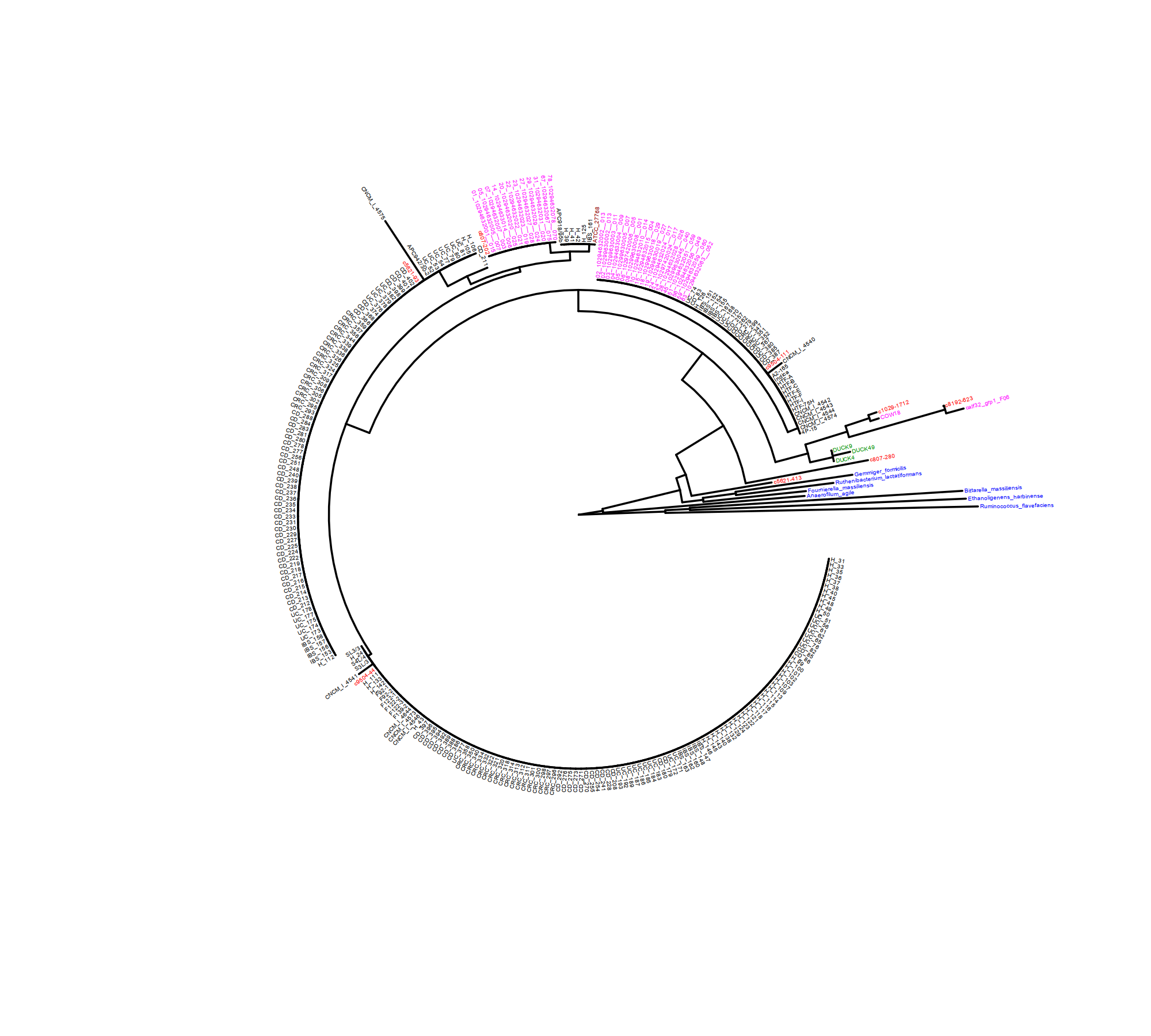

Supplement: S2 Fig — The evolutionary history was inferred using the Neighbor-Joining method after clustering the sequences with clustal Omega and plotting the tree after 1000 bootstrap replications. The type strain F. prausnitzii ATCC-27768 is shown in brown. F. prausnitzii sequences analysed were from human faeces (black), bovine faeces (magenta), duck faeces (green), milk centroids (red). Included were 7 sequences from non-Faecalibacterium members of the Ruminococcaceae family (blue). (TIF) [file pone.0221055.s002.tif]

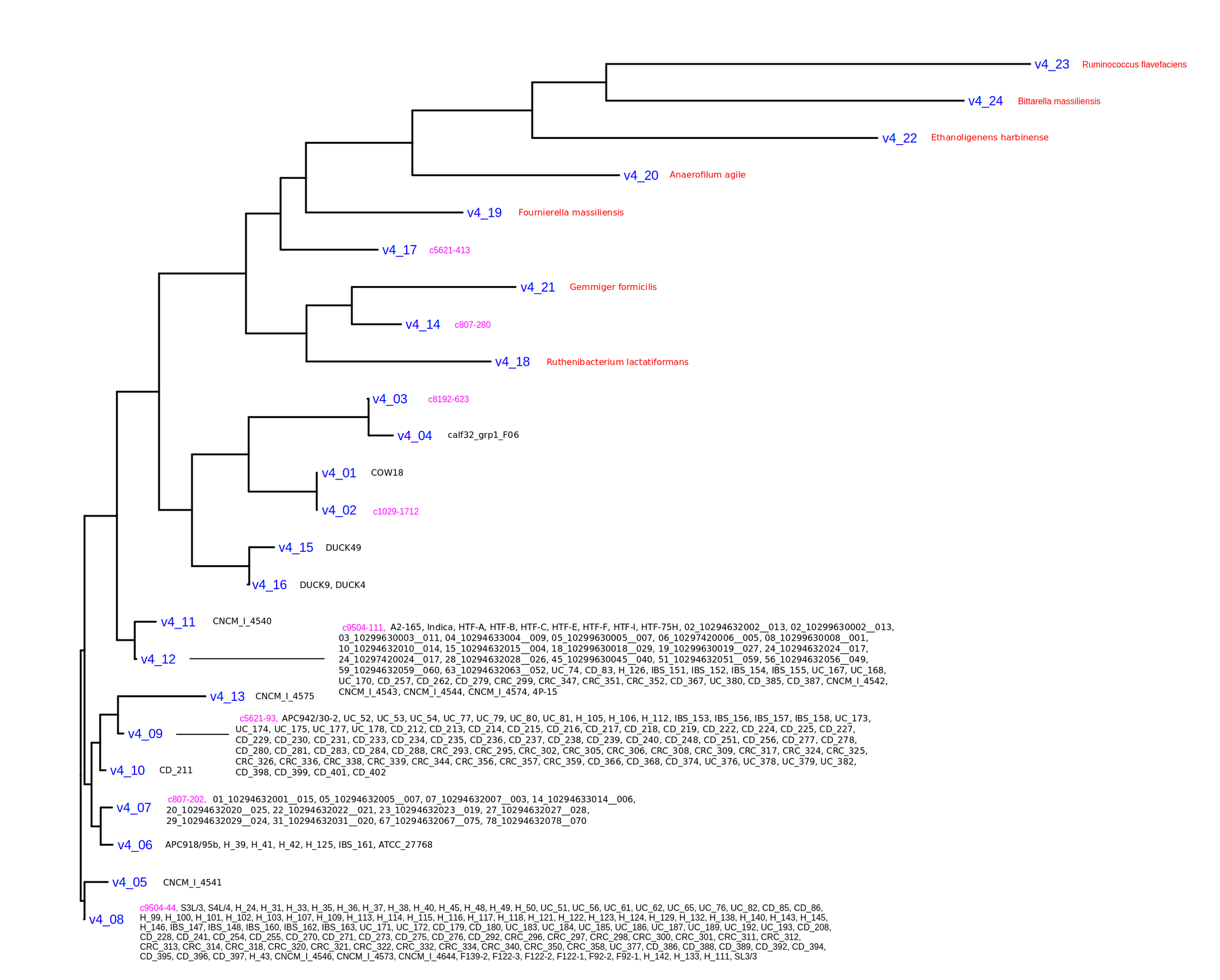

Supplement: S3 Fig — The evolutionary history was inferred using the Neighbor-Joining method after clustering the sequences with clustal Omega and plotting the tree after 100 bootstrap replications. The tree was additionally annotated to display the strains or species from which the dereplicated sequences (blue) derived. The names of the milk centroids are shown in magenta and the non-Faecalibacterium members of the Ruminococcaceae family are shown in red. (TIF) [file pone.0221055.s003.tif]
