# Supplementary material for: Faecalibacterium diversity in dairy cow milk
Source: PLoS One. 2019 Aug 16;14(8):e0221055. doi: 10.1371/journal.pone.0221055 (PMC6697359; doi:10.1371/journal.pone.0221055)
Supplement: S1 Table — Nineteen of the 21 cows were kept outdoors in a field growing pasture(ryegrass). In addition they were each fed grains, silage and hay. Cows 675 and 774 were kept indoors and fed on grains and hay. Diet figures are daily intake (pasture is estimated). (DOCX) [file pone.0221055.s004.docx]

**Supporting Information Table 1 : Diet**

| **sample** | **cow** | **Location** | **Pasture Approx (kg DM)** | **Wheat (kg DM)** | **Maize silage (kg DM)** | **Pasture silage** | **Lucerne Hay** | **Canola** |
| --- | --- | --- | --- | --- | --- | --- | --- | --- |
| msb001334 | 400 | field | 6-8kg | 6 kg | 4kg | 6kg |  |  |
| msb001335 | 604 | field | 6-8kg | 6 kg | 4kg | 6kg |  |  |
| msb001336 | 644 | field | 6-8kg | 6 kg | 4kg | 6kg |  |  |
| msb001337 | 659 | field | 6-8kg | 6 kg | 4kg | 6kg |  |  |
| msb001338 | 666 | field | 6-8kg | 6 kg | 4kg | 6kg |  |  |
| msb001339 | 675 | indoors |  | 9kg |  |  | 9kg | 1kg |
| msb001340 | 690 | field | 6-8kg | 6 kg | 4kg | 6kg |  |  |
| msb001342 | 774 | indoors |  | 9kg |  |  | 9kg | 1kg |
| msb001343 | 807 | field | 6-8kg | 6 kg | 4kg | 6kg |  |  |
| msb001344 | 854 | field | 6-8kg | 6 kg | 4kg | 6kg |  |  |
| msb001345 | 1029 | field | 6-8kg | 6 kg | 4kg | 6kg |  |  |
| msb001347 | 5601 | field | 6-8kg | 6 kg | 4kg | 6kg |  |  |
| msb001348 | 5621 | field | 6-8kg | 6 kg | 4kg | 6kg |  |  |
| msb001349 | 6808 | field | 6-8kg | 6 kg | 4kg | 6kg |  |  |
| msb001350 | 8192 | field | 6-8kg | 6 kg | 4kg | 6kg |  |  |
| msb001351 | 7905 | field | 6-8kg | 6 kg | 4kg | 6kg |  |  |
| msb001353 | 8421 | field | 6-8kg | 6 kg | 4kg | 6kg |  |  |
| msb001354 | 9501 | field | 6-8kg | 6 kg | 4kg | 6kg |  |  |
| msb001355 | 9504 | field | 6-8kg | 6 kg | 4kg | 6kg |  |  |
| msb001356 | 9510 | field | 6-8kg | 6 kg | 4kg | 6kg |  |  |
| msb001357 | 9536 | field | 6-8kg | 6 kg | 4kg | 6kg |  |  |

**Location and diet of cows**.

Nineteen of the 21 cows were kept outdoors in a field growing pasture(ryegrass). In addition they were each fed grains, silage and hay. Cows 675 and 774 were kept indoors and fed on grains and hay. Diet figures are daily intake (pasture is estimated).
