# Supplementary material for: Faecalibacterium diversity in dairy cow milk
Source: PLoS One. 2019 Aug 16;14(8):e0221055. doi: 10.1371/journal.pone.0221055 (PMC6697359; doi:10.1371/journal.pone.0221055)
Supplement: S2 Table — Read-pairs = total pairs of sequence reads generated by MiSeq; De-rep = number of unique 16s V4 sequences assembled by Pandaseq, filtered and de-replicated; OTU = number of different Greengenes OTU detected by MegaBlast with at least 97% identity and with >10 counts per million (cpm) abundance; Phyla = number of different phyla present at >10000cpm(1%) abundance; H = Shannon diversity Index; Chao1 = Chao1 species richness estimate. Shannon diversity Index and Chao1 species richness use the counts at species level as available in the August 2013 version of the Greengenes ribosomal RNA database, as are also the phyla and genera abundances. (DOCX) [file pone.0221055.s005.docx]

**Supporting Information Table 2 : DNA sequences summary**

| **Sample** | **cow** | **Read-pairs** | **De-rep** | **OTU** | **Phyla** | **H** | **Chao1** |
| --- | --- | --- | --- | --- | --- | --- | --- |
| msb001334 | 400 | 231370 | 61248 | 3478 | 6 | 4.23 | 746.71 |
| msb001335 | 604 | 283649 | 71685 | 2147 | 5 | 3.01 | 531.34 |
| msb001336 | 644 | 265184 | 48462 | 2008 | 6 | 2.87 | 544.45 |
| msb001337 | 659 | 288295 | 66922 | 1977 | 5 | 4.16 | 590.96 |
| msb001338 | 666 | 128528 | 32205 | 2293 | 6 | 4.51 | 564.08 |
| msb001339 | 675 | 242505 | 44563 | 2548 | 6 | 2.69 | 708 |
| msb001340 | 690 | 172883 | 45868 | 2884 | 6 | 4.16 | 705.13 |
| msb001342 | 774 | 255522 | 43976 | 1712 | 5 | 1.65 | 563.5 |
| msb001343 | 807 | 218586 | 53026 | 2339 | 5 | 3.74 | 549.02 |
| msb001344 | 854 | 308828 | 73864 | 2716 | 7 | 3.72 | 640.78 |
| msb001345 | 1029 | 306460 | 74503 | 2186 | 5 | 3.02 | 460.46 |
| msb001347 | 5601 | 220814 | 55933 | 3150 | 6 | 4.24 | 694.9 |
| msb001348 | 5621 | 217188 | 45790 | 2336 | 5 | 3.19 | 597.02 |
| msb001349 | 6808 | 246833 | 64729 | 3670 | 6 | 3.94 | 731.85 |
| msb001350 | 8192 | 240858 | 50374 | 1478 | 5 | 3.86 | 455.97 |
| msb001351 | 7905 | 503917 | 121271 | 3075 | 6 | 4.05 | 712.63 |
| msb001353 | 8421 | 259950 | 62079 | 2075 | 6 | 3.95 | 593.94 |
| msb001354 | 9501 | 297332 | 72937 | 2377 | 5 | 3.37 | 618.47 |
| msb001355 | 9504 | 254213 | 61909 | 3236 | 6 | 4.13 | 696.92 |
| msb001356 | 9510 | 308570 | 74147 | 2635 | 6 | 3.95 | 669.01 |
| msb001357 | 9536 | 267767 | 63828 | 2599 | 5 | 3.88 | 666.06 |

**Summary of sequencing and taxonomy analysis for each milk sample or cow**.

Read-pairs = total pairs of sequence reads generated by MiSeq; De-rep = number of unique 16s V4 sequences assembled by Pandaseq, filtered and de-replicated; OTU= number of different Greengenes OTU detected by MegaBlast with at least 97% identity and with >10 counts per million (cpm) abundance; Phyla = number of different phyla present at >10000cpm(1%) abundance; H = Shannon diversity Index; Chao1 = Chao1 species richness estimate. Shannon diversity Index and Chao1 species richness use the counts at species level as available in the August 2013 version of the Greengenes ribosomal RNA database, as are also the phyla and genera abundances.
