# Supplementary material for: Faecalibacterium diversity in dairy cow milk
Source: PLoS One. 2019 Aug 16;14(8):e0221055. doi: 10.1371/journal.pone.0221055 (PMC6697359; doi:10.1371/journal.pone.0221055)
Supplement: S3 Table — Faecalibacterium-related (from analysis using the Greengenes database) 16s v4 sequences, clustered using swarm2(see Fig 3) were compared to the Genbank Microbial 16s rRNA gene sequences database using megaBlast. Listed are results from the 6 most abundant centroid sequences, including centroid DNA sequence, Genbank accessions, expect values and sequence identities. (DOCX) [file pone.0221055.s006.docx]

**Supporting Information Table 3:**

**Top 6 cluster-centroids sequence and blast results (NCBI 16s Microbial)**

**>c9504-44_821**

CCTGTTTGCTACCCACACTTTCGAGCCTCAGCGTCAGTTGGTGCCCAGTAGGCCGCCTTCGCCACTGGTGTTCCTCCCGATATCTACGCATTCCACCGCTACACCGGGAATTCCGCCTACCTCTGCACTACTCAAGAAAAACAGTTTTGAAAGCAGTTCATGGGTTGAGCCCATGGATTTCACTTCCAACTTGTCTTCCCGCCTGCGCTCCCTTTACACCCAGTAATTCCGGACAACGCTTGTGACCTACGTT

**Accession Species/strain Expect-Value identity%**

NR_028961.1 Faecalibacterium prausnitzii strain ATCC-27768 3e-130 99%

NR_156911.1 Fournierella massiliensis strain AT2 3e-105 94%

NR_028997.1 Subdoligranulum variabile strain BI-114 7e-102 93%

NR_151900.1 Ruthenibacterium lactatiformans strain 585-1 3e-100 92%

NR_104846.1 Gemmiger formicilis strain X2-56 3e-100 92%

NR_029313.1 Anaerofilum pentosovorans strain Fae 2e-92 91%

NR_029315.1 Anaerofilum agile strain F 2e-92 91%

**>c9504-111_244**

CCTGTTTGCTACCCACACTTTCGAGCCTCAGCGTCAGTTGGTGCCCAGTAGGCCGCCTTCGCCACTGGTGTTCCTCCCGATATCTACGCATTCCACCGCTACACCGGGAATTCCGCCTACCTCTGCACTACTCAAGAAAAACAGTTTTGAAAGCAGTTTATGGGTTGAGCCCATAGATTTCACTTCCAACTTGTCTTCCCGCCTGCGCTCCCTTTACACCCAGTAATTCCGGACAACGCTTGTGACCTACGTT

**Accession Species/strain Expect-Value identity%**

NR_028961.1 Faecalibacterium prausnitzii strain ATCC-27768 7e-127 99%

NR_028997.1 Subdoligranulum variabile strain BI-114 3e-105 94%

NR_151900.1 Ruthenibacterium lactatiformans strain 585-1 2e-103 93%

NR_104846.1 Gemmiger formicilis strain X2-56 2e-103 93%

NR_156911.1 Fournierella massiliensis strain AT2 7e-102 93%

NR_029313.1 Anaerofilum pentosovorans strain Fae 5e-94 91%

NR_029315.1 Anaerofilum agile strain F 5e-94 91%

**>c5621-93_138**

CCTGTTTGCTACCCACACTTTCGAGCCTCAGCGTCAGTTGGTGCCCAGTAGGCCGCCTTCGCCACTGGTGTTCCTCCCGATATCTACGCATTCCACCGCTACACCGGGAATTCCGCCTACCTCTGCACTACTCAAGAAAAACAGTTTTGAAAGCAGTTCATGGGTTGAGCCCATGGATTTCACTTCCAACTTGTTCTCCCGCCTGCGCTCCCTTTACACCCAGTAATTCCGGACAACGCTTGTGACCTACGTT

**Accession Species/strain Expect-Value identity%**

NR_028961.1 Faecalibacterium prausnitzii strain ATCC-27768 7e-127 99%

NR_156911.1 Fournierella massiliensis strain AT2 3e-105 94%

NR_028997.1 Subdoligranulum variabile strain BI-114 3e-100 92%

NR_151900.1 Ruthenibacterium lactatiformans strain 585-1 2e-98 92%

NR_104846.1 Gemmiger formicilis strain X2-56 2e-98 92%

NR_029313.1 Anaerofilum pentosovorans strain Fae 2e-92 91%

NR_029315.1 Anaerofilum agile strain F 2e-92 91%

**>c807-202_63**

CCTGTTTGCTACCCACACTTTCGAGCCTCAGCGTCAGTTGGTGCCCAGTAGGCCGCCTTCGCCACTGGTGTTCCTCCCGATATCTACGCATTCCACCGCTACACCGGGAATTCCGCCTACCTCTGCACTACTCAAGAAAAACAGTTTTGAAAGCAGTTCATGGGTTGAGCCCATGGATTTCACTTCCAACTTGCTTTCCCGCCTGCGCTCCCTTTACACCCAGTAATTCCGGACAACGCTTGTGACCTACGTT

**Accession Species/strain Expect-Value identity%**

NR_028961.1 Faecalibacterium prausnitzii strain ATCC-27768 3e-130 99%

NR_156911.1 Fournierella massiliensis strain AT2 7e-107 94%

NR_028997.1 Subdoligranulum variabile strain BI-114 7e-102 93%

NR_151900.1 Ruthenibacterium lactatiformans strain 585-1 3e-100 92%

NR_104846.1 Gemmiger formicilis strain X2-56 3e-100 92%

NR_029313.1 Anaerofilum pentosovorans strain Fae 5e-94 91%

NR_029315.1 Anaerofilum agile strain F 5e-94 91%

**>c8192-623_79**

CCTGTTTGCTACCCACACTTTCGAGCCTCAGCGTCAGTTAGTGCCCAGTAGGCCGCCTTCGCCACTGGTGTTCCTCCCGATATCTACGCATTCCACCGCTACACCGGGAATTCCGCCTACCTCTGCACCACTCAAGAAGAGAAGTTTTGAAAGCAGTTTACGAGTTGAGCCCGTAGATTTCACTTCCAACTTTCTCTCCCGCCTGCGCTCCCTTTACACCCAGTAATTCCGGACAACGCTTGTGACCTACGTT

**Accession Species/strain Expect-Value identity%**

NR_028961.1 Faecalibacterium prausnitzii strain ATCC-27768 3e-110 95%

NR_156911.1 Fournierella massiliensis strain AT2 3e-100 92%

NR_151900.1 Ruthenibacterium lactatiformans strain 585-1 2e-98 92%

NR_028997.1 Subdoligranulum variabile strain BI-114 4e-95 91%

NR_104846.1 Gemmiger formicilis strain X2-56 2e-93 91%

NR_029313.1 Anaerofilum pentosovorans strain Fae 5e-89 90%

NR_029315.1 Anaerofilum agile strain F 5e-89 90%

**>c1029-1712_21**

CCTGTTTGCTACCCACACTTTCGAGCCTCAGCGTCAGTTAGTGCCCAGTAGGCCGCCTTCGCCACTGGTGTTCCTCCCGATATCTACGCATTCCACCGCTACACCGGGAATTCCGCCTACCTCTGCACCACTCAAGAAAGATAGTTTTGAAAGCAGTTTACGAGTTGAGCCCGTAGATTTCACTTCCAACTTATCTTCCCGCCTGCGCTCCCTTTACACCCAGTAATTCCGGACAACGCTTGTGACCTACGTT

**Accession Species/strain Expect-Value identity%**

NR_028961.1 Faecalibacterium prausnitzii strain ATCC-27768 2e-113 96%

NR_151900.1 Ruthenibacterium lactatiformans strain 585-1 3e-100 92%

NR_156911.1 Fournierella massiliensis strain AT2 8e-97 92%

NR_028997.1 Subdoligranulum variabile strain BI-114 2e-93 91%

NR_104846.1 Gemmiger formicilis strain X2-56 8e-92 91%

NR_029313.1 Anaerofilum pentosovorans strain Fae 5e-89 90%

NR_029315.1 Anaerofilum agile strain F 5e-89 90%
